# Preventive health care services utilization and its associated factors among older adults in rural communities in Anambra State, Nigeria

**DOI:** 10.11604/pamj.2021.39.83.26997

**Published:** 2021-05-28

**Authors:** Ifunanya Rosemary Obi, Kamtoochukwu Maduneme Obi, Eunice Nguungwan Seer-Uke, Samuel Ifeanyichukwu Onuorah, Nonye Peculiar Okafor

**Affiliations:** 1 Department of Physical and Health Education, Federal College of Education (Technical), Umunze, Nigeria,; 2 Aguata Diocesan Community Health Services, Diocese of Aguata, Ekwulobia, Anambra State, Nigeria,; 3 Department of Human Kinetics and Health Education, Benue State University, Makurdi, Nigeria,; 4 Department of Human Kinetics and Health Education, University of Nigeria, Nsukka, Nigeria,; 5 Department of Human Kinetics and Sports Studies, Alvan Ikoku College of Education, Owerri, Nigeria

**Keywords:** Preventive, health care, utilization, older adults, rural communities, Anambra State

## Abstract

**Introduction:**

quality of life and life expectancy of people are improved when preventive health care services are utilized because these identify treatable health problems and puts life-threatening diseases in check. Morbidity and mortality associated with age-related chronic disease among the older adults is on the increase, therefore, this study aims at determining preventive health care services utilization among older adults in rural communities in Anambra State.

**Methods:**

a cross sectional design adopted for this study was carried out on older adults from the ages of 65 years and above in rural communities in Anambra State from October 2019 to January 2020. Data were collected through researcher-administered structured questionnaire. Data were analysed using univariable and multivariable regression analysis.

**Results:**

a total of 1944 older adults participated with an overall cluster percentage of 40.6% older adults utilizing investigated preventive health care services. The results of the multivariable analysis indicates that the following factors were associated with utilization of preventive healthcare services: male gender (aOR: 0.443, 95%CI: 0.281 - 5.472, p=0.47), level of education; primary (aOR: 1.536, 95%CI: 1.201 - 5.261, p=0.00), secondary (aOR: 4.516, 95%CI: 3.192 - 6.242, p=0.00), and tertiary (aOR: 3.407, 95%CI: 3.199 - 5.666, p=0.00)], income of N50,000-N100,000 (aOR: 2.754, 95%CI: 1.066 - 10.766, p=0.01), and N100,000 and above (aOR: 4.233, 95%CI: 1.846 - 12.811, p=0.00)], and health insurance [aOR: 0.691, 95%CI: 0.422 - 1.945, p=0.03].

**Conclusion:**

preventive health care services were under-utilized. Creating awareness on the importance of utilizing preventive health care services is highly recommended since most age-related chronic diseases once established may last a lifetime and affect quality of life and wellbeing.

## Introduction

Preventive health care services are necessary measures undertaken by people to improve and protect their health and wellbeing. Quality of life and life expectancy of people are improved when preventive health care services are utilized because these identify treatable health problems and puts in check life-threatening diseases [1]. Examples of preventive services include immunizations, blood pressure screening, dietary counseling, chemoprophylaxis, health education, use of insecticides, alcohol and drug misuse counseling, depression counseling, cancer screening for example mammography and X-ray [2]. Health care utilization is rising all over the world. As the populations age, there is increase in health care utilization [3]. Globally, recent projections show that aging is the greatest driver of health care utilization because factors such as advancement in medicine, improved nutrition, control of infectious diseases has led to the decrease of death rate of persons advanced in years [4]. Under-utilization of preventive health care services has led to an increase in morbidities and mortalities associated with chronic disease [5]. High mortality rates of 21.7 percent occurred as a result of delay in health care use and this increase is the negative impacts of reduced health care utilization, especially in cases of chronic diseases [6,7]. Currently, less than 30% of populations in developed countries utilize preventive health care services even when the services are free [8-10]. In developing countries, majority of the population utilize health care when they are actually sick [11]. In the rural communities, demand-side barriers are present in health care facilities such as lack of human and material resources, bad roads and poor transport systems and this has led to under-utilization for health care goods and services [11].

Many people in low- and middle-income countries are unable to utilize health care because of socio-economic factors, cultural beliefs and practices, distance and most importantly problem of the health system itself such as availability, affordability and quality of care [12]. Older adults are affected by chronic diseases and under-utilization of preventive health care services will increase morbidity and mortality since most of these chronic diseases will not be identified and treated early. Many older adults in rural communities have challenges that have led to under-utilization of health care and older adults in rural communities in Anambra State, South East Nigeria may not be an exception. The rich in the society utilize health care for preventive, curative, rehabilitative and maintenance purposes while the poor use health care for curative purposes and most of the health care and health care services utilized by the rich is rarely found in the rural communities. Utilization of preventive health care services by older adults is important for preventing diseases, promoting good health and longevity. To design appropriate measures for improving preventive health care service utilization among older adults in rural communities, there is need for sufficient data especially for the study area. This paper is aimed at determining the preventive health care service utilization and its associated factors among older adults in rural communities in Anambra State, Nigeria.

## Methods

**Study design and setting:** a cross sectional design was adopted for this study and was carried out on older adults from the ages of 65 years and above in rural communities in Anambra State from October 2019 to January 2020. Anambra State is in South-Eastern Nigeria and is made up of seven urban local government areas and fourteen rural local government areas.

### Study population

**Target population:** all older adults residing in rural communities during the study period were eligible population for the study. 4 percent of people in Anambra State are aged 65 years and above [13]. Using 2016 population projection of Anambra State which was 5,527,809; older adults in the State was 221,112 and this is 4 percent of the State population. The population of older adults in rural communities in Anambra State could not be ascertained at the time of this study but using the report that about 50.48 percent of older adults reside in rural communities in Nigeria [14], about 111,617 older adults which is 50.48 percent of 221,112 older adult population in the State reside in the rural communities in Anambra State.

**Sampling methods:** the sample size was determined using the suggestion that when a population is 100,000 and above at 95 percent confidence level and 5 percent confidence interval, the sample size would be 381 and above [15]. To get a representation of the target population, 1,944 older adults which is above 381 was used for the study. A multistage sampling procedure was used to select 1,944 older adults from households in rural communities. The first stage was the selection of seven rural local government areas used for the study from the fourteen rural local government areas using a simple random sampling technique of balloting without replacement. The second stage was the selection of rural communities used for the study. There are 75 communities in these selected seven rural local government areas. Simple random sampling technique of balloting without replacement was used and 38 rural communities were selected making sure that each selected rural local government area was represented. The third stage was the selection of villages from the selected rural communities. There are 323 villages in these selected rural communities. Simple random sampling technique of balloting without replacement was used and 162 villages were selected and each selected rural community and local government area was represented. About fifty percent of villages, communities, and rural local government areas were chosen so as to ensure generalizability of the findings. The fourth stage was the selection of households from the 162 villages. In each selected village, systematic sampling technique was used to select households with older adults that were 65 years and above. This procedure was used to select twelve older adults (six males and six females) each from 162 villages to make up the sample size of 1,944 older adults (972 males and 972 females). A household consisted of people that lived under the same roof and shared meals. For the purpose of this study, older adults were considered members of a household if he or she has slept in that compound or apartment in the study area for at least six months before the study.

**Inclusion/exclusion criteria:** older adults aged 65 year and above who were resident in rural communities in Anambra State Nigeria for at least 6 months prior to the study, met in households selected and gave verbal consent to participate in the study. Older adults not resident in rural communities, or have been residents for less than 6 months were excluded.

**Data collection:** data were collected using a researcher-administered structured questionnaire. The questionnaire comprised of two sections. Section one included socio-demographic and health system factors such as age, gender, level of education, income, belonging to a health insurance and distance, and section two included utilization of preventive health care services such as chemoprophylaxis, immunizations, general medical checkup, laboratory investigations, cancer screening, blood pressure screening and health counseling. This instrument was developed after relevant literature was reviewed. Face validity were determined by five experts in the University of Nigeria, Nsukka and reliability of the questionnaire was established using the split half method. A reliability coefficient of 0.75 was obtained using Spearman-Brown formula. Utilization data was collected by self-reports therefore use of preventive health care services was within the last three months prior to the study to avoid recall bias. The researchers made use of research assistants who were workers in health centers in the selected communities. They were briefed on the purpose and contents of the questionnaire.

**Definitions:** utilization of preventive health care services was considered adequate when majority (at least 70%(n=1360)) of the older adults used the preventive health care services while preventive health care services was said to be under-utilized when the proportion of those who used the services was below 70% (n=1360).

**Statistical analysis:** the data collected were subjected to descriptive statistics to analyse the demographic characteristics of the respondents and the utilisation of preventive healthcare services. Univariable and multivariable regression were used to explore the factors associated with preventive health care utilization. At the end of the univariable regression analysis, variables with a p value of less than 0.2 were selected for inclusion in the multivariable regression analysis. The Statistical Package for Social Sciences, SPSS version 22 was used for data analysis.

**Ethical consideration:** before data collection, a permission letter was obtained by the authors from the University of Nigeria, Nsukka to the community heads. Older adults were informed about the purpose of the study, and were assured of the confidentiality and anonymity of information obtained from them. Verbal consent was also obtained before participation in the study.

## Results

**Socio-demographic characteristics of respondents:** one thousand nine hundred and forty-four (1944) older adults made up of 972 (50%) males and 972 (50%) females answered the research questions making a 100% response rate. 1044 (53.7%) were 65 - 74 years of age, 615 (31.6%) had secondary education as their maximum educational attainment, 888 (45.7%) earned below N50,000.00 in a year, 510 (26.2%) belonged to health insurance, and 1124 (57.8%) travel a distance of 5km and less to access health care services (Table 1).

**Table 1:** socio-demographic and other characteristics of respondents in rural communities in Anambra State

Characteristics	Frequency	Percentage (%)
**Age**		
65 - 74 years	1044	53.7
75 and above	900	46.3
Total	1944	100.0
**Gender**		
Male	972	50.0
Female	972	50.0
Total	1944	100.0
**Level of education**		
Tertiary	525	27.0
Secondary	615	31.6
Primary	502	25.8
No formal education	302	15.5
Total	1944	100.0
**Income in Naira (₦) in a year**		
Below ₦50,000	888	45.7
₦50,000 - ₦100,000	563	29.0
Above ₦100,000	493	25.4
Total	1944	100.0
**Belonging to health insurance**		
Yes	510	26.2
No	1434	73.8
Total	1944	100.0
**Distance**		
5km and less	1124	57.8
Above 5km	820	42.2
Total	**1944**	**100.0**

**Utilization of preventive health care services:** out of 1944 older adults, 1095 (56.3%) utilized chemoprophylaxis, 985 (50.7%) immunizations, 743 (38.2%) general medical checkup, 739 (38.0%) laboratory investigations, 722 (37.1%) cancer screening, 654 (33.6%) blood pressure screening, 592 (30.5%) health counseling (Table 2). Overall cluster percentage of 40.6% (n=789) older adults utilized preventive health care services. From this, cluster percentages of 41.4% (n=805) older adults were aged 75 years and above, 43.4% (n=844) were males, 50.6% (n=984) had a maximum of secondary education, 44.3% (n=861) earned above ₦100,000.00 in a year, 46.9% (n=912) belonged to health insurance schemes and 46.0% (n=894) travelled above 5km to access preventive health care services (Table 2).

**Table 2:** utilization of preventive health care services by respondents

Preventive health care services	Yes		No	
	Frequency	Percentage (%)	Frequency	Percentage (%)
Chemoprophylaxis (drugs to prevent diseases)	1095	56.3	849	43.7
Immunization	985	50.7	959	49.3
General medical checkup	743	38.2	1201	61.8
Laboratory investigations	739	38.0	1205	62.0
Cancer screening	722	37.1	1222	62.9
Blood pressure screening	654	33.6	1290	66.4
Health counseling	592	30.5	1352	69.5
Cluster (%) total		**40.6**		**59.4**

**Factors associated with preventive health care services utilization:** the results of the univariable analysis revealed that gender (p=0.04), level of education (p=0.00), income (p=0.00), and health insurance (p=0.01) were significantly associated with preventive health care services utilization. The results of the multivariable analysis also indicates that the male gender was associated with utilization of preventive healthcare services [aOR: 0.443, 95%CI: 0.281 - 5.472, p=0.47]. This means that females are 0.443 times likely to utilize preventive healthcare services than males. Since p>0.05, it also means that being female is not significantly associated with preventive healthcare services utilization. In the same vein, attending the primary, secondary and tertiary level of education was significantly associated with preventive healthcare services utilization [primary (aOR: 1.536, 95%CI: 1.201 - 5.261, p=0.00), secondary (aOR: 4.516, 95%CI: 3.192 - 6.242, p=0.00), tertiary (aOR: 3.407, 95%CI: 3.199 - 5.666, p=0.00)]. This means that people with primary education are 1.536 times likely to utilize preventing healthcare services, people with secondary education are 4.516 times likely to utilize preventive healthcare services while people with tertiary education are 3.407 times likely to utilize preventive healthcare services than those with no formal education. People with secondary level of education had the highest odds (aOR: 4.516) of utilizing preventive healthcare services.

Income of ₦50,000-₦100,000 (aOR: 2.754, 95%CI: 1.066 - 10.766, p=0.01), and ₦100,000 and above (aOR: 4.233, 95%CI: 1.846 - 12.811, p=0.00)] was significantly associated with utilization. Thus, people who earn between ₦50,000 and ₦100,000 are 2.754 times likely to utilize preventive healthcare services while people who earn from ₦100,000 and above are 4.233 times likely to utilize preventive healthcare services than those who earn below ₦50,000. People in the high income quartile (₦100,000 and above) have the highest odds of utilizing preventive healthcare services. Belonging to a health insurance scheme was significantly associated with preventive healthcare services utilization [aOR: 0.691, 95%CI: 0.422 - 1.945, p=0.03]. Thus, people with no health insurance scheme are 0.691 (less likely) times likely to utilize preventive healthcare services. However, p<0.05, implies that those with no health insurance are also significantly associated with preventive healthcare services utilization though they have lesser odds of doing so than those with health insurance. Distance (less than 5km) to a health facility was associated with preventive healthcare services utilization [aOR: 0.731, 95%CI: 0.159 - 2.284, p=0.32]. This indicates that people living far from health facilities (5km and more) are 0.7 times likely to utilize preventive healthcare services than those living less than 5km. Since p>0.05, this indicates that a distance of 5km and above is not significantly associated with preventive healthcare services utilization (Table 3).

**Table 3:** univariable and multivariable analysis on factors affecting utilization of preventive health care services

	Univariable analysis		Multivariable analysis	
	aOR (95% CI)	p-value	aOR (95% CI)	p-value
**Gender**				
Male	Ref	Ref	Ref	Ref
Female	0.789 (0.376 - 6.725)	0.04	0.443 (0.281 - 5.472)	0.47
**Age**				
65 -74 years	0.876 (0.746 - 1.980)	0.32		
75 and above	0.855 (0.050 - 2.306)	0.49		
**Level of education**				
No formal	Ref	Ref	Ref	Ref
Primary	1.743 (1.043 - 5.581)	0.00	1.536 (1.201 - 5.261)	0.00
Secondary	1.440 (1.137 - 5.515)	0.00	4.516 (3.192 - 6.242)	0.00
Tertiary	1.301 (1.111 - 5.465)	0.00	3.407 (3.199 - 5.666)	0.00
**Income**				
Below ₦50,000	Ref	Ref	Ref	Ref
₦50,000 - ₦100,000	3.309 (2.808 - 7.364)	0.00	2.754 (1.066 - 10.766)	0.01
Above ₦100,000	4.534 (2.509 - 7.193)	0.00	4.233 (1.846 - 12.811)	0.00
**Health insurance**				
Yes	Ref	Ref	Ref	Ref
No	1.714 (1.692 - 2.386)	0.01	0.691 (0.422 - 1.945)	0.03
**Distance**				
5km and less	Ref	Ref	Ref	Ref
Above 5km	0.913 (0.113 - 1.420)	0.17	0.731 (0.159 - 2.284)	0.32

aOR=adjusted odds ratio; CI=Confidence interval; Ref=Reference category

## Discussion

Use of a service is considered adequate when majority of the population utilizes such service but in this study, utilization of preventive health care service by the respondents was less than 50% (n=972). However, slightly above 50% of the respondents utilized some preventive services such as chemoprophylaxis 1095 (56.3%), and immunization 985 (50.7%). Although studies reported under-utilization of preventive health care services in rural communities [16,17], we aimed at finding out utilization of preventive health care services among older adults in rural communities in Anambra State, Nigeria. It was expected that there would be adequate utilization because it is known that a greater proportion of the population used for the study are usually burdened with age-related health problems. The finding from this study was higher than that from a previous study conducted in Edo State, Nigeria [18] which gave utilization rates for preventive health care services as 11.5% and 8% respectively. The variations observed may be as a result of differences in methodology and services investigated. Patterns of health care utilization vary. The association of age, gender, level of education, belonging to a health insurance, and distance from residence to health care facility with preventive health care service utilization were investigated. The results of the univariate analysis showed that age was not a significant factor in the utilization of preventive health care services. This finding disagrees with the study conducted in Jordan [19], which showed that there is increase in utilization as one advances in age. This means that the discrepancies found in utilization of preventive health care services could be as a result of other demographic or environmental factors.

Gender and level of education were significantly associated with preventive health care services utilization. The results of the univariate analysis indicates that males had a greater odd ratio of utilising preventive healthcare services than females. This means that a greater proportion of respondents who utilized preventive services were significantly males. This finding may be as a result of societal beliefs that the male respondents had more resources to utilize health services as household heads. This finding was at variance with previous studies conducted in China [1], Ghana [20] and Chile [8]. These studies reported that females were more vulnerable than males thus had more encounters with the health facility. Another study conducted in Austria [21] reported that there was no gender difference in utilization of preventive health care services. Higher education is generally linked with better health literacy and health outcomes which could be achieved through the use of preventive health services. A greater proportion of the respondents who had secondary level of education had higher odds ratio hence, utilized preventive health care than other levels. Although this group of respondents was more in number when compared to other levels of education, it was expected that people with tertiary education would utilize preventive health care more than those with other levels of education. Studies [1,22] have also linked high educational attainment with high level of awareness thereby increasing the use of preventive health care services but this does not agree with this study. This could be due to the fact that high awareness breeds higher knowledge, improved living conditions and healthier lifestyles thereby reducing the odds of seeking preventive health care.

The study also found a statistically significant association of income with preventive health care utilization. A greater proportion of respondents reported an income of below N50,000 in a year, therefore the impact of cost on preventive health care is a challenge to its use. Studies have shown that low-income families tend to spend a greater part of their income on basic daily needs such as food, shelter and clothing than preventive health service because in most cases, the older adults are vulnerable to poverty and are dependent on others [1,22,23]. These reports are in agreement with the finding from this study which showed that a greater proportion of the respondents that utilized preventive health care services earned above N100,000 in a year. This means that the use of preventive health care services may not be a burden when added to other basic needs. Belonging to a health insurance is seen as a critical factor in preventive health service use; this was not the case in this study. A lesser proportion of respondents who were insured did not utilize preventive health care services. Studies show that adequate insurance coverage was lacking in rural communities and out of pocket payments for health care which is largely practiced in rural communities is a hindrance to health care use [1,24], it was expected that those respondents who were insured would adequately utilize preventive health care services. Another study opined that being insured does not adequately promote the use of preventive health care service because some of the services are not utilized even when they are free [1]. The authors were unable to ascertain whether the health insurance schemes were designed to cover basic preventive health care services.

Distance to health care facilities was significantly associated with preventive health care service utilization. This study discovered that a greater proportion of respondents resided close to health facilities and this did not positively affect utilization. This could be as a result of transport problems seen in rural communities such as poor or bad road network, or lack of good transportation systems. There is need to further investigate reasons for under-utilization of services even when the health facilities are near. A study conducted in Tanzania is at variance with this study, they reported that most of their respondents resided above 5km from health care facility and this led to under-utilization by their respondents [24,25]. The authors acknowledge some limitation of the study; data were collected from older adults in rural communities and secondary data source on preventive health care service utilization from health facilities in the study area were not obtained and this may limit generalization of findings of this study.

## Conclusion

This study revealed that a greater proportion of older adults in rural communities under-utilized preventive health care services. The factors associated with preventive health care utilization include gender, level of education, income, health insurance and distance to health facility. This implies that males with secondary level of education, who had no insurance, who earn a higher income and reside closer to health facilities are more likely to utilize preventive healthcare. There is need to educate both young and old on the importance of utilizing preventive health care services. This will create awareness and knowledge of these preventive health care services.

### What is known about this topic

- Previous studies show that developing countries under-utilize preventive health care services;
- Ageing is associated with chronic health problems, utilization of preventive health care services by older adults prevents diseases, identifies life-threatening health problem early and promotes longevity.

### What this study adds

- This study confirms the under-utilization of preventive health care services in rural communities;
- The study identified gender, level of education, income, health insurance and distance to be significantly associated with preventive health care utilization among older adults in rural communities;
- Awareness on the importance and availability of preventive health care services will improve its utilization.
